# Rho-associated protein kinase 2 (ROCK2): a new target of autoimmunity in paraneoplastic encephalitis

**DOI:** 10.1186/s40478-017-0447-3

**Published:** 2017-05-29

**Authors:** Stoyan Popkirov, Ilya Ayzenberg, Stefanie Hahn, Jan Bauer, Yvonne Denno, Nicole Rieckhoff, Christiane Radzimski, Volkmar H. Hans, Sebastian Berg, Florian Roghmann, Joachim Noldus, Christian G. Bien, Sabine Skodda, Jörg Wellmer, Winfried Stöcker, Christos Krogias, Ralf Gold, Uwe Schlegel, Christian Probst, Lars Komorowski, Ramona Miske, Ingo Kleiter

**Affiliations:** 1 Department of Neurology, Ruhr-Epileptology, Universitätsklinikum Knappschaftskrankenhaus Bochum, Ruhr University Bochum, In der Schornau 23-25, 44892 Bochum, Germany; 2 Department of Neurology, St. Josef Hospital Bochum, Ruhr University Bochum, Bochum, Germany; 3 Institute of Experimental Immunology, Euroimmun AG, Lübeck, Germany; 4 Department of Neuroimmunology, Center for Brain Research, Medical University Vienna, Vienna, Austria; 5 Institute of Pathology, Ruhr University Bochum, Bochum, Germany; 6 Department of Urology, Marien Hospital Herne, Ruhr University Bochum, Bochum, Germany; 7 Epilepsy Centre Bethel, Krankenhaus Mara, Bielefeld, Germany; 8 Department of Neurology, Knappschaftskrankenhaus Bochum, Ruhr University Bochum, Bochum, Germany

**Keywords:** Paraneoplastic encephalitis, Status epilepticus, Autoantibody, Rho-associated protein kinase 2, Urogential cancer

## Abstract

Onconeural antibodies are associated with cancer and paraneoplastic encephalitis. While their pathogenic role is still largely unknown, their high diagnostic value is undisputed. In this study we describe the discovery of a novel target of autoimmunity in an index case of paraneoplastic encephalitis associated with urogenital cancer.

A 75-year-old man with a history of invasive bladder carcinoma 6 years ago with multiple recurrences and a newly discovered renal cell carcinoma presented with seizures and progressive cognitive decline followed by super-refractory status epilepticus. Clinical and ancillary findings including brain biopsy suggested paraneoplastic encephalitis. Immunohistochemistry of the brain biopsy was used to characterize the inflammatory response. Indirect immunofluorescence assay (IFA) was used for autoantibody screening. The autoantigen was identified by histo-immunoprecipitation and mass spectrometry and was validated by expressing the recombinant antigen in HEK293 cells and neutralization tests. Sera from 125 control patients were screened using IFA to test for the novel autoantibodies.

IFA analysis of serum revealed a novel autoantibody against brain tissue. An intracellular enzyme, Rho-associated protein kinase 2 (ROCK2), was identified as target-antigen. ROCK2 was expressed in affected brain tissue and archival bladder tumor samples of this patient. Brain histopathology revealed appositions of cytotoxic CD8^+^ T cells on ROCK2-positive neurons. ROCK2 antibodies were not found in the sera of 20 patients with bladder cancer and 17 with renal cancer, both without neurological symptoms, 49 healthy controls, and 39 patients with other antineuronal autoantibodies. In conclusion, novel onconeural antibodies targeting ROCK2 are associated with paraneoplastic encephalitis and should be screened for when paraneoplastic neurological syndromes, especially in patients with urogenital cancers, occur.

**Electronic supplementary material:**

The online version of this article (doi:10.1186/s40478-017-0447-3) contains supplementary material, which is available to authorized users.

## Introduction

Paraneoplastic encephalitis is a rare disease that commonly presents with seizures, cognitive and psychiatric disturbances and is secondary to various tumors [9]. It usually affects limbic areas, but may also involve extralimbic structures. Paraneoplastic encephalitis is associated with neural antibodies targeting intracellular antigens or cell-surface proteins [6]. The former presumably are not pathogenic themselves, but are considered an epiphenomenon of the T cell-mediated immunoreaction against the antigen. As such, they are valuable diagnostic indicators of associated and sometimes occult cancer. In a recent multinational database study, only 1.2% of cases of paraneoplastic neurologic syndromes were related to kidney or bladder tumors [8]. Paraneoplastic encephalitis, in particular, has been associated with urological cancers only in very few cases [27]. Herein we report a unique case of biopsy-confirmed encephalitis associated with antineuronal autoantibodies against a hitherto unknown antigen in a patient with bladder and kidney tumors.

## Materials and methods

Reagents were obtained from Merck, Darmstadt, Germany or Sigma-Aldrich, Heidelberg, Germany if not specified otherwise.

### Patients

The index patient was treated for symptomatic epilepsy and later refractory status epilepticus due to paraneoplastic encephalitis at the Ruhr-University of Bochum, Germany. He died before paraneoplastic etiology of encephalitis was confirmed; written informed consent to publish the clinical course was obtained from his widow. Serum samples of 20 patients with bladder cancer and 17 patients with renal cancer without neurological disorders as well as of 49 healthy controls and 39 patients with other known antineuronal autoantibodies (anti-NMDAR, anti-Hu, anti-Yo, anti-Ri, anti-AQP4, anti-LGI1, anti-CASPR2, anti-GAD65) were tested as controls. Patient recruitment was prospective and written informed consent was obtained from each patient. This study was approved by the Ethics Committee of the Ruhr-University Bochum, Germany.

### Immunohistochemistry of brain biopsy

**Fig. 1:**
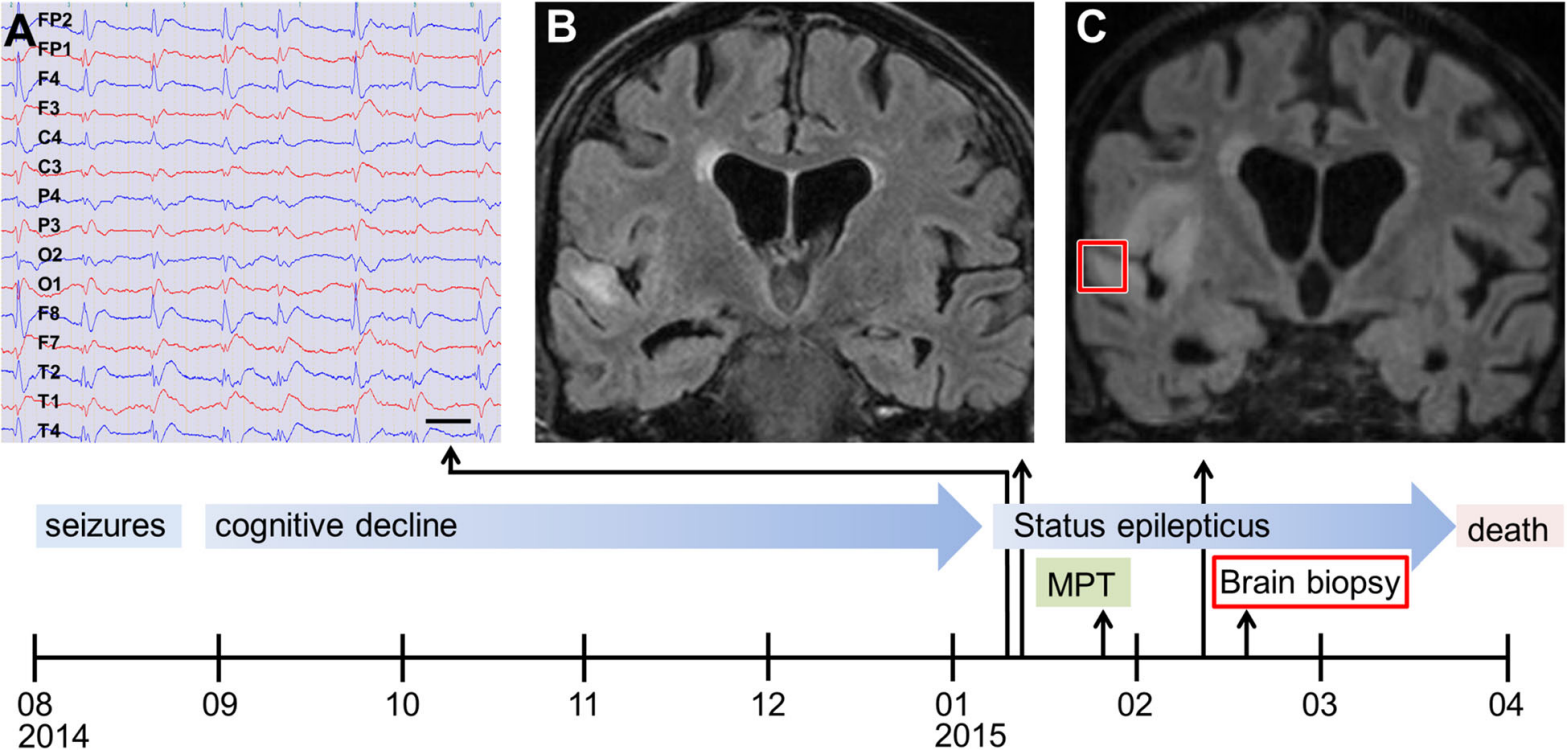
Clinical course, EEG and MRI of the index patient. Time axis of disease development with main symptoms, treatments and findings. **a** EEG excerpt in average potential reference montage shows right-sided periodic lateralized epileptiform discharges (PLED); bar: 1 s. **b** Coronal T2-FLAIR MRI on admission shows hyperintensity in the right superior temporal gyrus, compatible with inflammation. **c** Coronally reconstructed 3D-FLAIR MRI shows progression after 30 days. Abbreviations: MPT, methylprednisolone pulse therapy; T2-FLAIR, T2-weighted fluid attenuation inversion recovery; 3D-FLAIR, three dimensionally acquired fluid attenuation inversion recovery. Red square indicates site of brain biopsy

A biopsy of affected brain tissue was carried out to aid in the differential diagnosis (see Results section and Fig. 1 for details). Paraffin sections containing formalin fixed brain biopsy material were stained for lymphocyte markers CD3, CD8 and granzyme B (GrB), for CD68 recognizing macrophages and microglia as well as for immunoglobulin deposits and C9neo, the terminal complex of the complement cascade. Neurons were detected by NeuN. Terminal deoxynucleotidyl transferase-mediated dUTP nick-end labeling (TUNEL) was used to detect apoptotic cells. After autoimmunity against Rho-associated protein kinase 2 (ROCK2) was identified, we also stained for ROCK2 (HPA007459, Sigma-Aldrich). Confocal fluorescent triple stainings were done with NeuN/CD8/GrB and ROCK2/CD3/GrB. All stainings were performed as described elsewhere [4]; see Additional files 1, 2, 3, 4 for details.

### Indirect immunofluorescence assay

IFA was conducted using slides with a biochip array of brain tissue cryosections (hippocampus of rat, cerebellum of rat and monkey) combined with recombinant HEK293 cells separately expressing 27 different brain antigens (EUROIMMUN AG, Luebeck, Germany) as described previously [28]. Additionally, a standard biochip mosaic containing frozen sections of 30 different human, monkey and rat organs and tissues (EUROIMMUN AG, Luebeck, Germany) was applied to determine an autoantibody profile. Each biochip mosaic was incubated with 70 μL of PBS-diluted sample at room temperature for 30 min, washed with PBS-Tween and immersed in PBS-Tween for 5 min. In the second step, either fluorescein isothiocyanate (FITC)-labelled goat anti-human IgG (EUROIMMUN AG, Luebeck, Germany) or, for the determination of IgG subclasses, murine FITC labelled monoclonal antibodies against human IgG1, IgG2, IgG3, or IgG4 (Sigma-Aldrich, Taufkirchen, Germany), were applied and incubated at room temperature for 30 min. Slides were washed again with a flush of PBS-Tween and then immersed in PBS-Tween for 5 min. Slides were embedded in PBS-buffered, DABCO containing glycerol (approximately 20 μL per field) and examined by fluorescence microscopy. Cell nuclei were visualized by DNA staining with TO-PRO3 Iodide (dilution 1:2000) (ThermoFisher Scientific, Schwerte, Germany). Positive and negative controls were included. Samples were classified as positive or negative based on fluorescence intensity of the transfected cells in direct comparison with non-transfected cells and control samples. Endpoint titers refer to the last dilution showing visible fluorescence.

In some experiments, a polyclonal antibody against ROCK2 (HPA007459, Sigma-Aldrich, Taufkirchen, Germany) was used in the first step followed by incubation with anti-rabbit IgG-Cy3 (Jackson Research, Cambridgeshire, UK). Results were evaluated by two independent observers using a laser scanning microscope (LSM700; Zeiss, Jena, Germany). In competitive inhibition experiments, recombinant antigens were mixed with diluted serum sample 30 min before the IFA as described previously [30].

### Immunoprecipitation and identification of the antigen

Cerebellum from rat was dissected and shock-frozen in liquid nitrogen. The tissues were homogenized in solubilization buffer (100 mmol/L tris-HCl pH 7.4, 150 mmol/L sodium chloride, 2.5 mmol/L ethylenediamine tetraacetic acid, 0.5% (w/v) sodium deoxycholate, 1% (w/v) Triton X-100) containing protease inhibitors (Complete mini, Roche Diagnostics, Penzberg, Germany) with a Miccra D-8 (Roth, Karlsruhe, Germany) and a hand homogenizer (Sartorius, Göttingen, Germany) at 4 °C. The tissue lysates was centrifuged at 21,000 x g at 4 °C for 15 min and clear supernatants were incubated with patient’s serum (diluted 1:33) at 4 °C overnight. The samples were then incubated with Protein G Dynabeads (ThermoFisher Scientific, Schwerte, Germany) at 4 °C for 3 h to capture immunocomplexes. The beads were washed 3 times with PBS, and eluted with NuPage LDS sample buffer (ThermoFisher Scientific, Schwerte, Germany) containing 25 mmol/L dithiothreitol at 70 °C for 10 min, followed by SDS-PAGE (NuPAGE, ThermoFisher Scientific, Schwerte, Germany). Separated proteins were either visualized with Coomassie Brillant Blue (G-250) (Merck, Darmstadt, Germany) gel staining, and identified by mass spectrometric analysis or electrotransferred onto a nitrocellulose membrane by tank blotting with transfer buffer (ThermoFisher Scientific, Schwerte, Germany) according to the manufacturer’s instructions. The membranes were blocked with Universal Blot Buffer plus (EUROIMMUN AG, Luebeck, Germany) for 15 min and incubated with the polyclonal antibody against ROCK2 (HPA007459, Sigma-Aldrich, Taufkirchen, Germany, dilution 1:4000) in Universal Blot Buffer plus for 3 h, followed by 3 washing steps with Universal Blot Buffer (EUROIMMUN AG, Luebeck, Germany), a second incubation for 30 min with anti-rabbit-IgG-AP (Jackson Research, Cambrigeshire, UK), 3 washing steps, and staining with NBT/BCIP substrate (EUROIMMUN AG, Luebeck, Germany).

### Mass spectrometry

Mass spectrometry sample preparation was performed as reported by Koy et al. [19]. See Additional files 1, 2, 3, 4 for details.

### Recombinant expression of ROCK2 in HEK293 cells

The coding DNA for human ROCK2 (UNIPROT acc. #O75116) was obtained by PCR on commercially available cDNA (codes for half of the protein, IRCMp5012C0731D, Imagenes, Nottingham, UK) and primers (ATACGTCTCTAGATATGACATACCAACTAAAAGTTATAC and ATACGTCTCCTCGAGTTAGCTAGGTTTGTTTGGGGCAAGC) as well as gene synthesis. The amplification products and DNA fragments obtained from gene synthesis were digested with Esp3I and ligated with pTriEx-1 (Merck, Darmstadt, Germany) after digestion with NcoI and XhoI. ROCK2 was expressed in the human cell line HEK293 after ExGen500-mediated transfection (ThermoFisher Scientific, Schwerte, Germany) according to the manufacturer’s instructions.

For the preparation of substrates for the IFA, HEK293 were grown on sterile cover glasses, transfected, and allowed to express ROCK2 for 48 h. Cover glasses were washed with PBS, fixed with acetone for 10 min at room temperature, air-dried, cut into millimeter-sized fragments (biochips) and used as substrates in IFA as described. Alternatively, cells were transfected in standard T-flasks and the cells were harvested after 48 h. The cell suspension was centrifuged at 1,500 x g, 4 °C for 20 min and the resulting sediment was extracted with 20 mmol/L tris-HCl pH 7.4, 50 mmol/L potassium chloride, 5 mmol/L ethylenediamine tetraacetic acid. The extracts were stored in aliquots at -80 °C until further use.

### Histopathology of renal and bladder tumors

Formalin-fixed paraffin-embedded renal and bladder carcinoma and, as controls, tumor-free areas from the index patient tissue were sectioned (4 μm). Slices were placed onto slides, deparaffinized, rehydrated, and subjected to heat-induced epitope-retrieval using Target Retrieval Solution (pH 9, 3-in-1, Dako, Hamburg, Germany) according to the supplier’s instructions. Subsequently, the slides were washed with Tris buffered saline (TBS) containing 0.05% Tween-20 at room temperature. Blocking was performed with Serum-free Protein Block (Thermo Fisher Scientific, Schwerte, Germany) for 10 min. Polyclonal rabbit anti-ROCK2 (HPA007459, Sigma-Aldrich, Taufkirchen, Germany) was diluted 1:250 in Dako antibody diluent and then applied for 30 min. As a negative control, rabbit immunoglobulin fraction (X0936, Dako, Hamburg, Germany) was used. Incubation was performed using the Bond Polymer Refine Detection system (Leica Biosystems, Wetzlar, Germany) according to the manufacturer’s instructions. 3,3′-Diaminobenzidine (DAB; Leica Biosystems, Wetzlar, Germany) development was stopped after 10 min. Hematoxylin (Leica Biosystems, Wetzlar, Germany) was used for counterstaining. Slides were mounted with Neo-Mount, water-free mounting medium (VWR, Darmstadt, Germany).

## Results

### Clinical course and imaging of the index patient

In August 2014 a 75-year-old man was admitted due to several episodes of confusion and aphasia. Medical history included arterial hypertension, multiple renal cysts and infiltrating urothelial carcinoma of the bladder 6 years ago with multiple recurrences and eventual radical cystoprostatectomy, pelvic lymphadenectomy and neobladder reconstruction 4 years ago. The patient was in complete remission afterwards, but did not attend oncological follow-up. Brain magnetic resonance imaging (MRI) was unremarkable besides subcortical microangiopathic lesions. Cerebrospinal fluid (CSF) analysis showed autochthonous oligoclonal bands in CSF indicating intrathecal immunoglobulin (Ig)G synthesis, but was otherwise normal. Electroencephalography (EEG) revealed right temporal spike-and-wave discharges and levetiracetam was started. The patient was discharged with the diagnosis of symptomatic epilepsy, most likely due to microangiopathic lesions.

Over the next 6 months he developed a progressive cognitive decline followed by an acute deterioration with agitation, somnolence and gait disturbance. In January 2015 he was readmitted with hyperkinetic delirium and multifocal myoclonic and ballistic movements, as well as grasping movements suggesting either visual hallucinations or seizure-related automatisms. These symptoms did not respond to intravenous antiepileptic (4 mg lorazepam, 2 g levetiracetam) and antipsychotic (15 mg haloperidol) treatment. Intubation anesthesia was necessary to enable further workup. CSF showed slightly elevated protein (69,9 mg/dl) and autochthonous oligoclonal bands in CSF indicating intrathecal IgG synthesis. EEG showed right-sided periodic lateralized epileptiform discharges (PLED) (Fig. 1a). On brain MRI FLAIR signal changes without contrast enhancement in the right temporal lobe and insular cortex were evident (Fig. 1b). Serum and CSF were negative for antibodies against the common neural antigens (see Additional files 1, 2, 3, 4 for details). Further laboratory tests for infectious, autoimmune, metabolic or neoplastic disease were unremarkable. Abdominal computed tomography (CT) revealed renal cysts and a renal mass deemed non-suspect. Paraneoplastic or autoimmune encephalitis was suspected and steroid treatment given (1 g methylprednisolone intravenously daily for 7 days with tapering off). The patient developed a super-refractory status epilepticus that persisted despite treatment with levetiracetam, lacosamide, clobazam, phenytoin and deep anesthesia with midazolam and propofol (verifiable burst-suppression for more than 24 h). Despite steroid treatment, follow-up brain MRI revealed disease progression with additional signal changes in the insular cortex (Fig. 1c); thus, rare infectious causes were reconsidered. Exhaustive workup including left temporal brain biopsy failed to identify an infectious agent. Brain biopsy was resected openly from the left superior temporal gyrus (approximately 1x1x1 cm) using neuronavigation to retrieve suspicious tissue identified by MRI (Fig. 1c). After routine neuropathological work-up, histopathology results were consistent with either viral or autoimmune T cell mediated encephalitis.

The patient developed staphylococcal septicemia and was treated accordingly. Repeat thoracic and abdominal contrast enhanced CT scan now revealed the mass on the left kidney to be highly suspect and showed enlarged local lymph nodes. Radical nephrectomy was performed; histology confirmed low grade (G2) papillary renal cell carcinoma. Due to the overall morbidity with recurrent infections and persistent status epilepticus, further tumor staging and therapy were deferred. The non-convulsive status epilepticus eventually remitted under high-dose phenytoin and the addition of lorazepam and the patient could be taken off the ventilator. Due to the considerable morbidity and poor prognosis, treatment was de-escalated in accordance with the wish of the family. The patient died 3 weeks later. Autopsy was not granted.

### Immunohistochemistry of brain biopsy

**Fig. 2:**
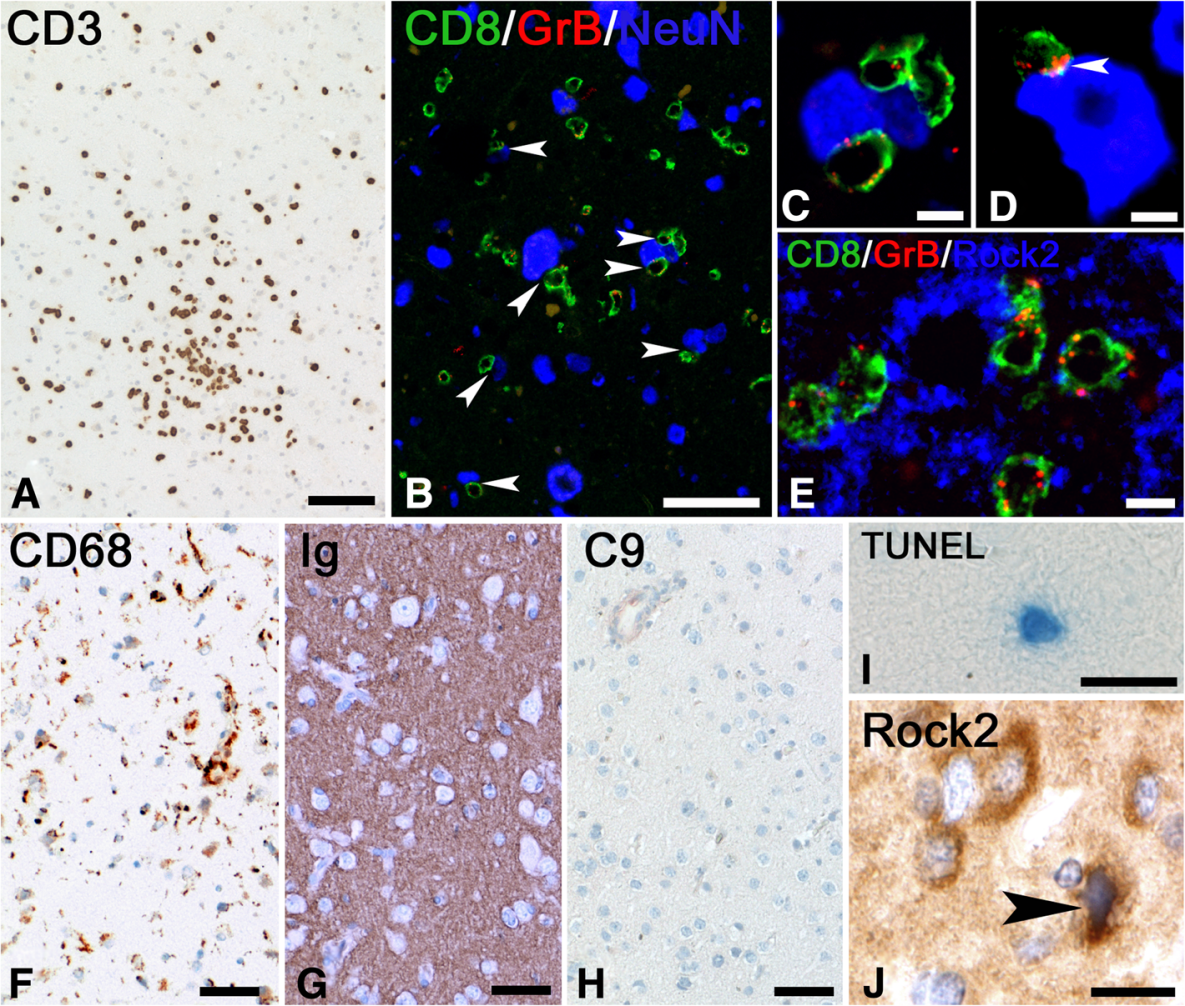
Immunohistochemistry of brain biopsy suggesting T cell-mediated inflammation. **a** Staining for CD3 shows the presence of large numbers of T cells in the parenchyma. Bar: 10 μm. **b** Confocal triple staining for CD8 (*green*), GrB (*red*) and NeuN (*blue*) shows multiple appositions (*arrowheads*) of cytotoxic T cells on neurons. Bar: 20 μm. **c** and **d** show the same triple staining for CD8 (*green*), GrB (*red*) and NeuN (*blue*) as (**b**). The staining in (**c**) shows that multiple CD8^+^/GrB^+^ T cells can be found in apposition to neurons. The staining in (**d**) again shows GrB translocation (*arrowhead*), now facing the neuronal membrane. Bars: 5 μm. **e** Triple staining for CD8 (*green*), GrB (*red*) and ROCK2 (*blue*) reveals that neurons with appositions of T cells are Rock2 positive. Bar: 20 μm. **f** Staining for CD68 shows moderate microglia activation. Bar: 5 μm. **g** Staining for anti-human-Ig shows staining in the parenchyma as a sign of immunoglobulin leakage. Bar: 20 μm (**h**) Staining for C9neo fails to reveal parenchymal staining. Bar: 50 μm. **i** TUNEL stain reveals the presence of single degenerating cells in the parenchyma. Bar: 20 μm. **j** Staining for ROCK2 shows the presence of multiple ROCK2^+^ neurons. The neuron indicated by the arrowhead shows a condensed nucleus suggesting apoptosis. Bar: 10 μm

Staining for CD3 showed the presence of large numbers of T lymphocytes in the meninges, perivascular space of blood vessels and in the parenchyma (Fig. 2a). Many of the CD3^+^ T cells were also positive for the CD8^+^ cytotoxic T cell subset as well as for the cytotoxic granule marker granzyme B. Quantification of cells in the parenchyma showed that 168.4 CD3^+^ T cells/mm^2^ were present. 87% of these T cells were also positive for CD8 (146.8 CD8^+^ T cells/mm^2^) while 50% of these T cells also were positive for Granzyme-B (85.6 cells/mm^2^). Appositions of such cytotoxic T cells to NeuN^+^ (Fig. 2b-d) and ROCK2^+^ neurons (Fig. 2e), suggesting specific T cell attacks, could be demonstrated. Staining for CD68 showed moderate microglia activation (Fig. 2f) at a density of 171.2 CD68^+^ cells/mm^2^. In addition, the presence of immunoglobulin and complement deposition was analyzed. Although a diffuse staining for immunoglobulins was seen, no neurons with uptake of immunoglobulins were detected (Fig. 2g). In addition we did not find any deposition of the terminal complex (C9neo) of the complement cascade (Fig. 2h). Finally, to investigate if neuronal cell death is present, sections were stained for TUNEL. Indeed some apoptotic cells were found in the parenchyma (Fig. 2i). In addition, staining for ROCK2 showed some positive neurons with condensed nuclei suggesting apoptosis (Fig. 2j).

### Detection of antineuronal autoantibodies

**Fig. 3:**
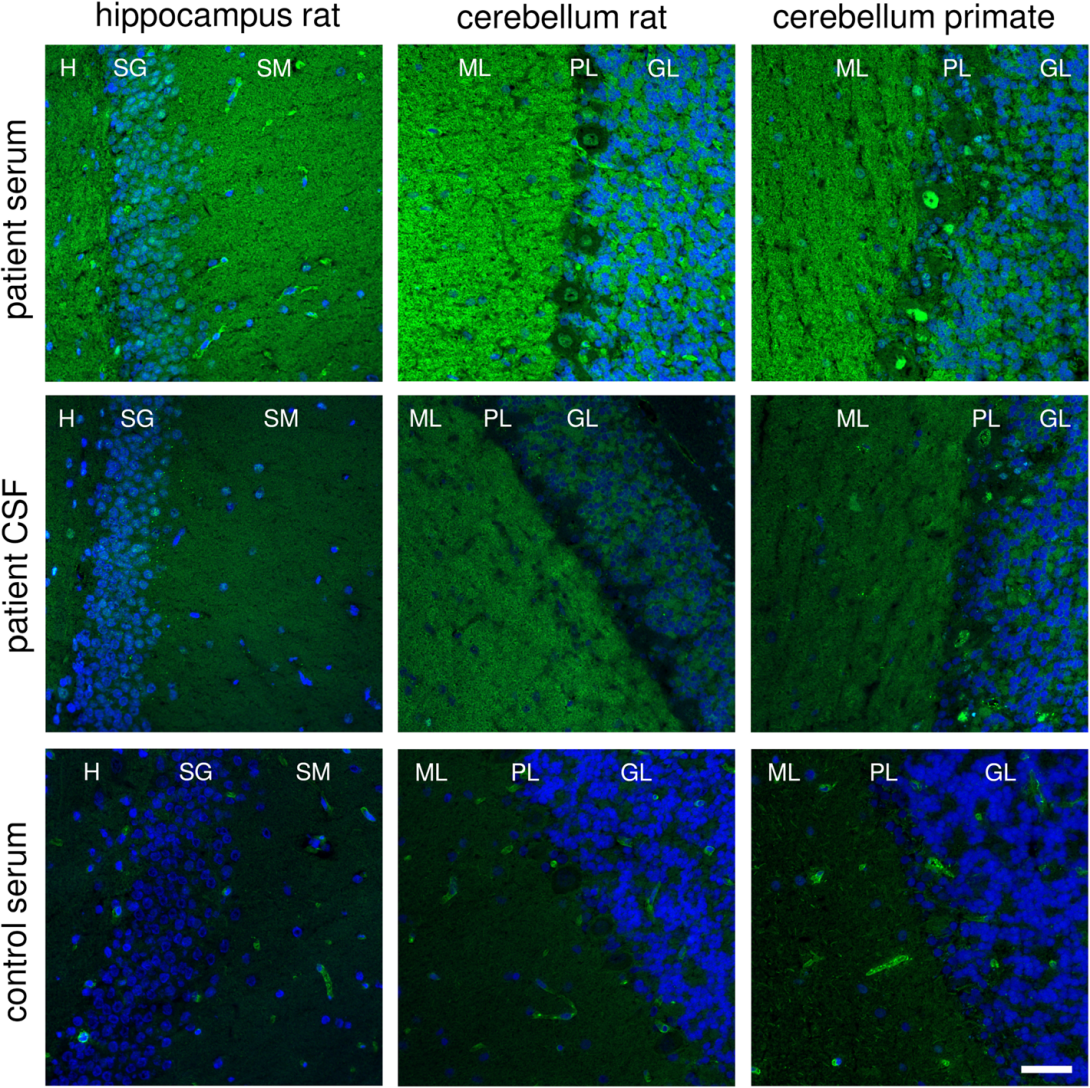
Immunofluorescence staining of central nervous system tissue. Cryosections were incubated with patient’s serum, control serum (each 1:32) or patient’s CSF (undiluted) in the first step, and with Alexa488-labelled goat anti-human immunoglobulin G (*green*) in the second step. Nuclei were counterstained by incubation with TO-PRO-3 iodide (*blue*). A fine-granular to homogeneous staining of molecular layer of both rat and monkey cerebellum as well as rat hippocampus and plaster-like staining of cerebellum granular layer excluding Purkinje cells was obtained. Scale bar = 50 μm; all figures same magnification. H = hilus, SM = stratum moleculare, SG = stratum granulosum, ML = molecular layer, PL = Purkinje cell layer, GL = granule cell layer

IFA analysis of the patient’s serum and CSF showed a fine-granular to homogeneous staining of the molecular layer of both rat and monkey cerebellum as well as of rat hippocampus and a plaster-like staining of the cerebellum granular layer at a titer of 1:320 (serum) and 1:1.6 (CSF) (Fig. 3). No staining of cerebellar Purkinje cells was observed. A similar pattern was observed with the undiluted patient’s CSF (Fig. 3). Autoantibodies were of the IgG class. Further analyses were conducted with recombinant HEK293 cells expressing 27 established neuronal human autoantigens: Hu, Yo, Ri, CV2, PNMA2, ARHGAP26, ZIC4, DNER/Tr, GAD65, GAD67, amphiphysin, recoverin, GABA_B_ receptor, GABA_A_ receptor, glycine receptor, DPPX, IgLON5, glutamate receptors (types NMDA, AMPA, mGluR1, mGluR5), LGI1, CASPR2, aquaporin 4 (M1 and M23 isoform), GluRD2, and myelin oligodendrocyte glycoprotein. However, none revealed reactivity (data not shown).

### Identification of Rho-associated protein kinase 2 as the target antigen

**Fig. 4:**
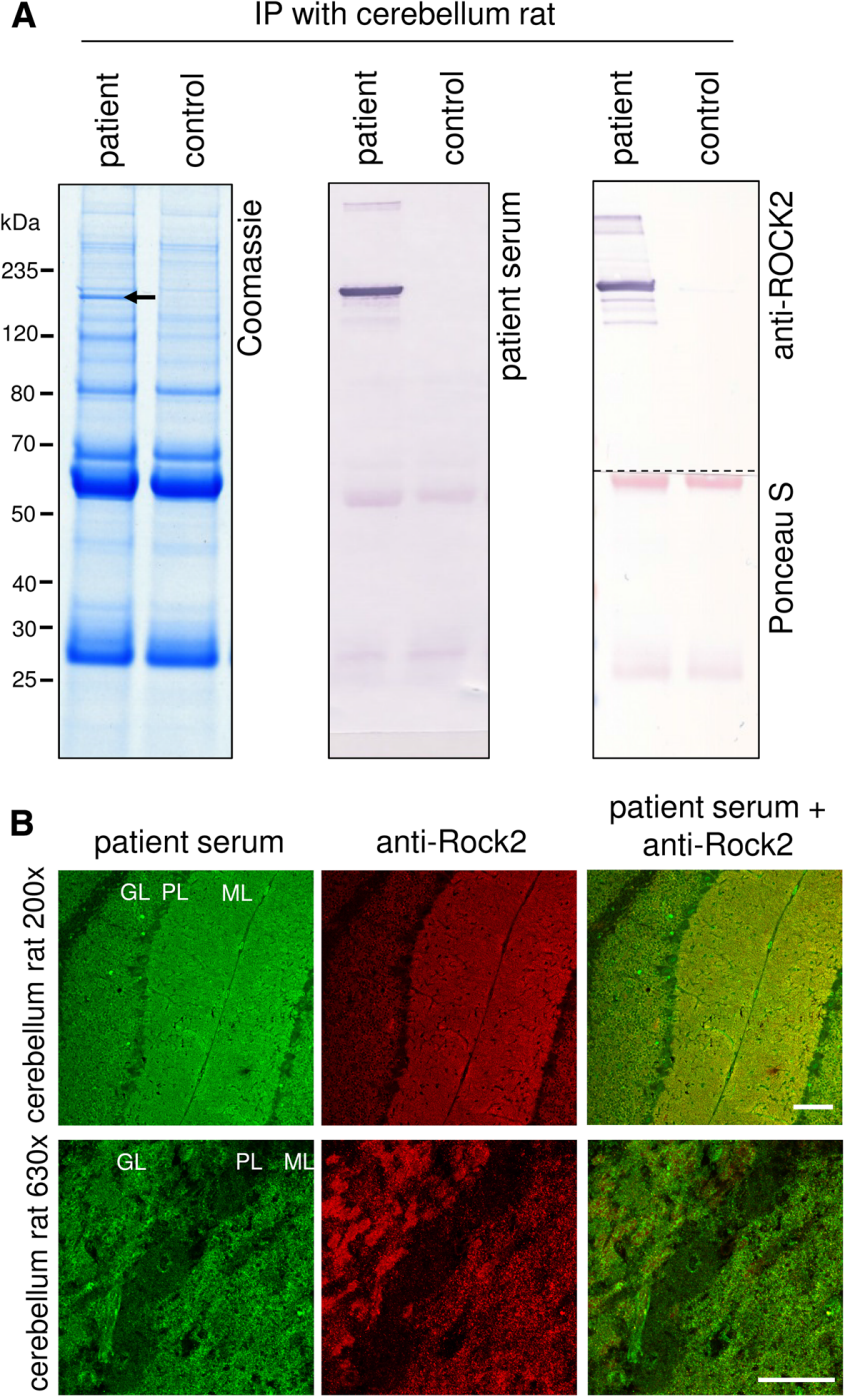
Identification of Rho-associated protein kinase 2 as the target antigen. **a** Lysates of rat cerebellum were incubated with patient or control sera (1:33). Immunocomplexes were isolated with protein-G-coated magnetic beads, eluted by SDS and subjected to SDS-PAGE analysis followed by (*left*) staining with colloidal Coomassie, (*middle*) Western blot using patient serum or (*right*) Western blot using polyclonal rabbit anti-ROCK2 and Ponceau S staining. Arrow indicates the position of the immunoprecipitated antigen at about 160 kDa. **b** Double immunofluorescence staining of cerebellar tissues with patient serum (1:50, *green*) and rabbit anti-ROCK2 antibody (1:250, *red*). The anti-ROCK2 antibody produced fluorescence patterns matching those generated by the patient’s serum. Scale bar = 100 μm ML = molecular layer, PL = Purkinje cell layer, GL = granule cell layer

Compared to healthy controls an additional protein of approximately 160 kDa was detected in SDS-PAGE of immunoprecipitates (Fig. 4a). Using mass spectrometry, the 160-kDa protein was identified as ROCK2. Western blot analysis of the immunoprecipitate using a polyclonal anti-ROCK2 antibody or patient serum revealed a strong reaction at 160 kDa, which was absent in the similarly prepared controls (Fig. 4a). When used in IFA, the commercial anti-ROCK2 antibody produced fluorescence patterns on cerebellum matching those generated by the patient’s serum (Fig. 4b).

**Fig. 5:**
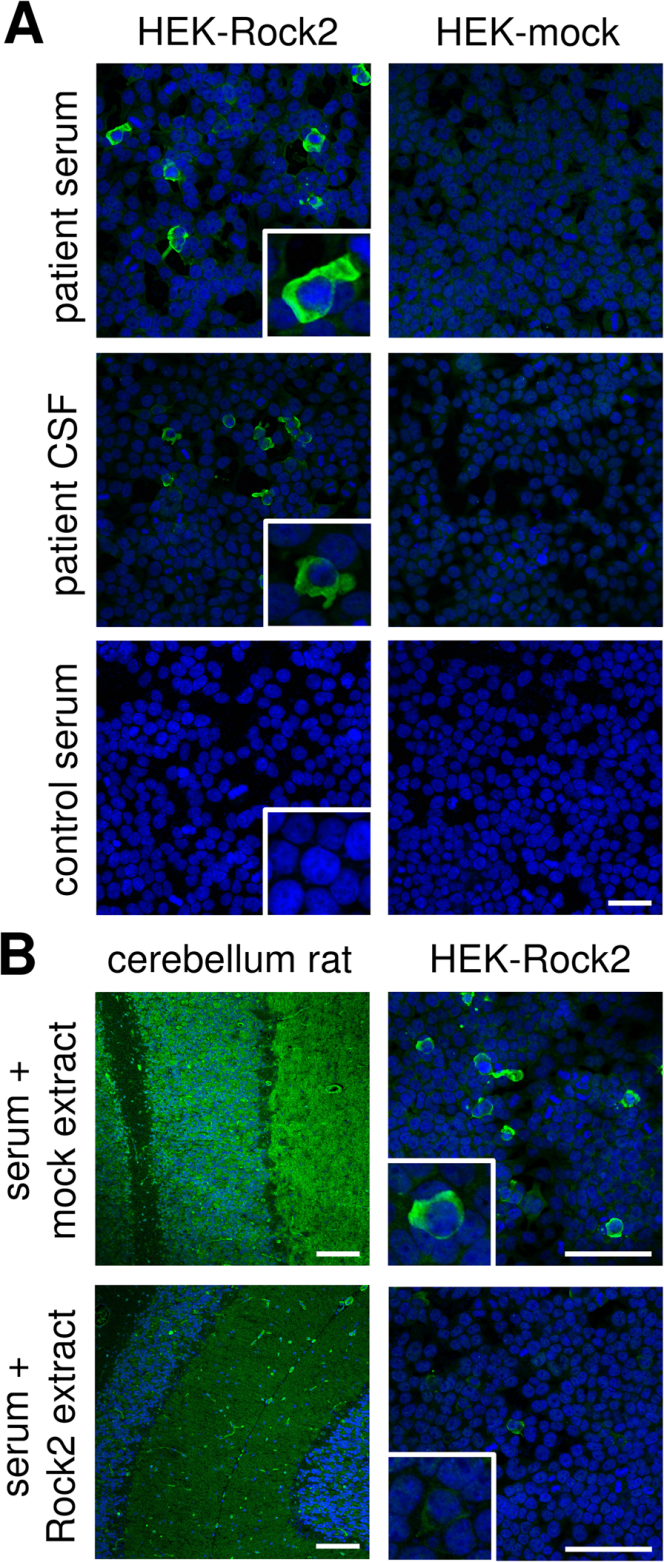
Verification of ROCK2 as the novel autoantigen by indirect immunofluorescence. **a**: Indirect immunofluorescence using acetone-fixed ROCK2- or mock-transfected HEK293 cells incubated with patient’s serum, control serum (each 1:320) or patient’s CSF (1:10) (*green*). Scale bar = 50 μm; all figures same magnification. **b**: Neutralisation of immunofluorescence reaction on cerebellum rat and ROCK2-transfected HEK293 cells. Patient serum (*green*) was pre-incubated with extracts of HEK293 cells transfected with ROCK2 or with empty vector as control. The extract containing ROCK2 abolished the immune reaction. Nuclei were counterstained by incubation with TO-PRO-3 iodide (*blue*). Inserts show enlargement of positive and negative ROCK2-transfected cells. Scale bar = 100 μm

As a proof for correct antigen identification, the patient samples were then tested by IFA using transfected HEK293 cells, which expressed ROCK2 (Fig. 5a). Strong staining was obtained on cells expressing ROCK2 at a titer of 1:32000 (serum) and 1:320 (CSF). The ROCK2 antibody index of 1.57 was not indicative of local antibody synthesis in the CSF. However, specific intrathecal antibody production is not obligatory in paraneoplastic syndromes, especially in case of intracellular antigens and T-cells mediated inflammation and damage. Analysis of the IgG subclass distribution revealed an IgG2 > IgG1 reactivity. ROCK2 as the patient’s autoantibody target was further confirmed by competitive blocking of antibody binding to brain tissue by prior incubation of HEK293 fractions containing ROCK2 (Fig. 5b).

### Expression of ROCK2 in tumor biopsies from the index patient

Formalin-fixed, paraffin-embedded sections of patient bladder and renal tumors as well as tumor-free healthy control tissue from the same surgical specimens were immunohistochemically stained with a polyclonal anti-ROCK2 antibody. Strong cytoplasmic anti-ROCK2 reactivity was observed in normal bladder epithelium as well as in bladder carcinoma (Additional file 1: Figure S1A). In contrast, anti-ROCK2 showed only weak staining in normal glomerular Bowman’s epithelium and proximal tubule epithelium whereas no reactivity was evident in kidney carcinoma (Additional file 1: Figure S1B).

### Disease specificity of anti-ROCK2 autoantibodies

Sera from healthy controls (*n* = 49), patients with various neuronal autoantibodies (*n* = 39), and patients with bladder (*n* = 20) or renal carcinoma (*n* = 17), both without neurological disease (see Additional file 2: Table S1 in Additional file 3: Supplementary Materials for a summary of clinical data), were analyzed by IFA in parallel to the samples of the index patient. None of these control sera produced a similar immunofluorescence pattern on the different brain tissues or showed a reaction with the recombinant ROCK2 substrate.

## Discussion

Paraneoplastic neurological disorders, and especially encephalitis, are exceptional in urological malignancies [27]. Only six cases of paraneoplastic limbic encephalitis associated with renal cancer have been described so far [5, 7, 10, 17, 20, 22, 33]. In bladder cancer, this association appears to be even rarer [24, 27]. Remarkably, there have been no reports of autoantibody detection in any of those cases. Paraneoplastic encephalitis was suspected in our patient based on the clinical syndrome with subacute cognitive deterioration and refractory seizures, the hyperintense temporal MRI lesions and the history of urological cancer. This diagnosis was corroborated only post mortem by the detection of neuronal autoimmunity and the findings of brain biopsy.

The detected antineuronal antibodies bound to the molecular layer of rat hippocampus and both molecular and granular layer of the cerebellum on rat and monkey sections. Using mass spectrometry ROCK2 was identified as the intracellular target antigen. This finding was confirmed using ROCK2-recombinant HEK293 cells and a neutralisation test. Immunohistochemical staining against ROCK2 revealed an intensive expression of the antigen in infiltrating urothelial carcinoma of the bladder in our patient, making the paraneoplastic nature of ROCK2 antibodies likely. In support of this, ROCK2 autoantibodies were not identified in the sera of any of the 37 control patients with bladder or renal carcinoma. ROCK2 antibodies were also not found in any of the healthy controls and in the controls with other antineuronal antibodies.

Neuropathology revealed apposition of granzyme B^+^ cytotoxic T cells to neurons, which is also found in paraneoplastic encephalitis associated with “classic” intracellular onconeural antibodies [3]. Moreover, these appositions where found with ROCK2^+^ neurons. Stainings for immunoglobulin deposits and complement activation were negative, indicating that no antibody-mediated response occurred, as can be seen in encephalitis with antibodies against surface antigens like anti-LGI1 or anti-CASPR2 [18]. TUNEL staining, assessing acute cellular degeneration, revealed some neuronal damage. Similar findings were reported in encephalitis with anti-Hu or anti-Ma2 antibodies, especially when the biopsy or autopsy material was collected later in the disease course [6]. Staining for ROCK2 revealed positive neurons with signs of apoptosis.

The neuropathology findings in the present case show many features of paraneoplastic encephalitis associated with antibodies against other intracellular antigens, and the main pathogenic mechanism is probably cytotoxic T cell attack [3]. Direct pathogenicity of ROCK2-antibodies is unlikely, as it would require that they pass the blood brain barrier and the cell membrane of the target cells before binding ROCK2 and disabling or changing its function. However, since there is still some debate surrounding a possible pathogenetic role of onconeural antibodies [3], it is worthwhile to consider ROCK2’s function in both tumor and brain.

ROCK2 is one of two mammalian homologs of Rho-associated coiled-coil containing kinases and is expressed intracellularly [15]. It is expressed in neurons, muscle cells as well as in kidney and bladder epithelium [13, 15]. ROCK2 has been implicated in many cellular functions, including actin organization, cell migration, neuronal growth cone guidance, synaptic transmission as well as cancer cell invasion and proliferation [13, 15]. In a number of cancers (breast, bladder, liver, melanoma and others) the ROCK2 pathway is implicated in the metastatic process [21, 25]. In both bladder and renal cancers, an elevation of ROCK2 expression has been associated with tumor invasion, metastasis, and an unfavorable prognosis [2, 16]. Moreover, the ROCK2 inhibitor Fasudil has been shown to suppress cell proliferation and migration of urothelial cancer cells [1].

Recently, the ROCK pathway has been identified as a constituent of neuronal regeneration and degeneration [11]. In neurons, enhanced intrinsic ROCK-activity critically disrupts the growth cone machinery, hampering regenerative processes. Furthermore, microglial activation of ROCK signaling maintains the pro-inflammatory M1-phenotype in favor of the anti-inflammatory M2-state. These and other effects (reviewed in Hensel et al. [11]) help explain how ROCK-inhibition can enhance axonal regeneration and counteract neuroinflammatory processes in neurodegenerative diseases such as Parkinson’s Disease [31, 32] or Alzheimer’s Disease [29], and in autoimmune disorders such as Multiple Sclerosis [12]. Interestingly, ROCK-inhibition has also shown ameliorating effects in animal models of epilepsy [14]. Nevertheless, assuming a direct effect of ROCK2-antibodies on neural dysfunction and brain pathology in the present case would be highly speculative.

ROCK2 is not the only intracellular kinase targeted by antibodies in autoimmune encephalitis. The BR serine/threonine kinase 2 has been identified as a target autoantigen in limbic encephalitis and small cell lung carcinoma [26] and the adenylate kinase 5 has been found in patients with autoimmune encephalitis without known cancer [23]. In both cases T-cell mediated inflammation has been proposed to underly the encephalitis process [23, 26].

## Conclusion

In conclusion, ROCK2-autoantibodies are a novel candidate biomarker for paraneoplastic encephalitis associated with urological cancer. Their epidemiology, pathogenicity and diagnostic value should be addressed in future studies.

## Additional files

Additional file 1: Figure S1. Immunohistochemical staining of the patient’s tumor tissue. Immunohistochemical staining with polyclonal rabbit anti-ROCK2 antibody (1:250) reveals strong reactivity in the patient’s invasive bladder carcinoma as well as in normal tumor free urothelium (A). However, ROCK2 shows an only weak staining in normal glomerular Bowman’s epithelium and in proximal tubule epithelium whereas expression is absent from patient’s papillary renal cell carcinoma (B). Rabbit immunoglobulin control stainings are negative, as expected. (scale bar = 100 μm; all figures same magnification). (TIF 13993 kb)

Additional file 2: Table S1. Patients with urological cancers whose sera were screened for anti-ROCK2 antibodies. (DOCX 14 kb)

Additional file 3: Supplementary Methods. Immunohistochemistry of brain biopsy; Mass spectrometry. Supplementary Results: Routine neural antibody testing. (DOCX 19 kb)

Additional file 4: Figure S2. Control stainings for Ig and C9neo. (A) Staining for Ig in control brain shows some reactivity in bloodvessels (arrowheads). Bar: 50 μm. (B) Staining for C9neo shows no reactiity in the brain. Bar: 50 μm. (C) Staining for Ig in the brain of an NMO patient shows strong deposition of Ig around a bloodvessel. Bar: 50 μm (D) Staining for C9neo, in this case shows deposition around a bloodvessel. (TIF 4965 kb)

## References

[1] Abe H, Kamai T, Hayashi K (2014). The Rho-kinase inhibitor HA-1077 suppresses proliferation/migration and induces apoptosis of urothelial cancer cells. BMC Cancer.

[2] Abe H, Kamai T, Tsujii T (2008). Possible role of the RhoC/ROCK pathway in progression of clear cell renal cell carcinoma. Biomed Res.

[3] Bauer J, Bien CG (2016). Neuropathology of autoimmune encephalitides. Handb Clin Neurol.

[4] Bauer J, Lassmann H (2016). Neuropathological techniques to investigate central nervous system sections in multiple sclerosis. Methods Mol Biol.

[5] Bell BB, Tognoni PG, Bihrle R (1998). Limbic encephalitis as a paraneoplastic manifestation of renal cell carcinoma. J Urol.

[6] Bien CG, Vincent A, Barnett MH (2012). Immunopathology of autoantibody-associated encephalitides: clues for pathogenesis. Brain.

[7] Blanc P, Martoia R, Louvet C, Bequet D, Laurens A (1988). Cancer sur rein unique et syndromes paranéoplasiques multiples (hypertension artérielle, polyglobulie, hypercalcémie, encéphalopathie limbique). Rev Med Interne.

[8] Giometto B, Grisold W, Vitaliani R, Graus F, Honnorat J, Bertolini G, Euronetwork PNS (2010). Paraneoplastic neurologic syndrome in the PNS Euronetwork database: a European study from 20 centers. Arch Neurol.

[9] Gultekin SH, Rosenfeld MR, Voltz R, Eichen J, Posner JB, Dalmau J (2000). Paraneoplastic limbic encephalitis: neurological symptoms, immunological findings and tumour association in 50 patients. Brain.

[10] Harrison JW, Cherukuri R, Buchan D (2015). Renal Cell Carcinoma Presenting with Paraneoplastic Hallucinations and Cognitive Decline from Limbic Encephalitis. J Gen Intern Med.

[11] Hensel N, Rademacher S, Claus P (2015). Chatting with the neighbors: crosstalk between Rho-kinase (ROCK) and other signaling pathways for treatment of neurological disorders. Front Neurosci.

[12] Hou SW, Liu CY, Li YH (2012). Fasudil ameliorates disease progression in experimental autoimmune encephalomyelitis, acting possibly through antiinflammatory effect. CNS Neurosci Ther.

[13] Iizuka M, Kimura K, Wang S (2012). Distinct distribution and localization of Rho-kinase in mouse epithelial, muscle and neural tissues. Cell Struct Funct.

[14] Inan S, Büyükafşar K (2008). Antiepileptic effects of two Rho-kinase inhibitors, Y-27632 and fasudil, in mice. Br J Pharmacol.

[15] Julian L, Olson MF (2014). Rho-associated coiled-coil containing kinases (ROCK): structure, regulation, and functions. Small GTPases.

[16] Kamai T, Tsujii T, Arai K (2003). Significant association of Rho/ROCK pathway with invasion and metastasis of bladder cancer. Clin Cancer Res.

[17] Kararizou E, Markou I, Zalonis I (2005). Paraneoplastic limbic encephalitis presenting as acute viral encephalitis. J Neurooncol.

[18] Körtvelyessy P, Bauer J, Stoppel CM (2015). Complement-associated neuronal loss in a patient with CASPR2 antibody-associated encephalitis. Neurol Neuroimmunol Neuroinflamm.

[19] Koy C, Mikkat S, Raptakis E (2003). Matrix-assisted laser desorption/ionization-quadrupole ion trap-time of flight mass spectrometry sequencing resolves structures of unidentified peptides obtained by in-gel tryptic digestion of haptoglobin derivatives from human plasma proteomes. Proteomics.

[20] Kujawa KA, Niemi VR, Tomasi MA, Mayer NW, Cochran E, Goetz CG (2001). Ballistic-choreic movements as the presenting feature of renal cancer. Arch Neurol.

[21] Loirand G (2015). Rho Kinases in Health and Disease: From Basic Science to Translational Research. Pharmacol Rev.

[22] Newman NJ, Bell IR, McKee AC (1990). Paraneoplastic limbic encephalitis: neuropsychiatric presentation. Biol Psychiatry.

[23] Ng AS, Kramer J, Centurion A, Dalmau J, Huang E, Cotter JA, Geschwind MD (2015). Clinico-pathological correlation in adenylate kinase 5 autoimmune limbic encephalitis. J Neuroimmunol.

[24] Richardson EP, Hedley-Whyte ET (1985). Case records of the Massachusetts General Hospital. Weekly clinicopathological exercises. Case 30-1985. A 52-year-old woman with a progressive neurologic disorder and a pelvic mass. N Engl J Med.

[25] Rodriguez-Hernandez I, Cantelli G, Bruce F, Sanz-Moreno V (2016) Rho, ROCK and actomyosin contractility in metastasis as drug targets. F1000Research 5(F1000 Faculty Rev):783 10.12688/f1000research.7909.1 PMC4856114 27158478

[26] Sabater L, Gómez-Choco M, Saiz A, Graus F (2005). BR serine/threonine kinase 2: a new autoantigen in paraneoplastic limbic encephalitis. J Neuroimmunol.

[27] Sacco E, Pinto F, Sasso F, Racioppi M, Gulino G, Volpe A, Bassi P (2009). Paraneoplastic syndromes in patients with urological malignancies. Urol Int.

[28] Scharf M, Miske R, Heidenreich F (2015). Neuronal Na+/K+ ATPase is an autoantibody target in paraneoplastic neurologic syndrome. Neurology.

[29] Song Y, Chen X, Wang LY, Gao W, Zhu MJ (2013). Rho kinase inhibitor fasudil protects against β-amyloid-induced hippocampal neurodegeneration in rats. CNS Neurosci Ther.

[30] Stöcker W, Otte M, Ulrich S (1987). Autoimmunity to pancreatic juice in Crohn’s disease: results of an autoantibody screening in patients with chronic inflammatory bowel disease. Scand J Gastroenterol Suppl.

[31] Tatenhorst L, Eckermann K, Dambeck V (2016). Fasudil attenuates aggregation of α-synuclein in models of Parkinson’s disease. Acta Neuropathol Commun.

[32] Tönges L, Frank T, Tatenhorst L (2012). Inhibition of rho kinase enhances survival of dopaminergic neurons and attenuates axonal loss in a mouse model of Parkinson’s disease. Brain.

[33] Vital C, Escourolle R, Hauw JJ, Rivel J (1973). L’encéphalite limbique paranéoplasique. Étude anatomo-clinique de deux observations. Arch Anat Pathol (Paris).

