# Supplementary figures and images for: Rho-associated protein kinase 2 (ROCK2): a new target of autoimmunity in paraneoplastic encephalitis
Source: Acta Neuropathol Commun. 2017 May 29;5:40. doi: 10.1186/s40478-017-0447-3 (PMC5448146; doi:10.1186/s40478-017-0447-3)

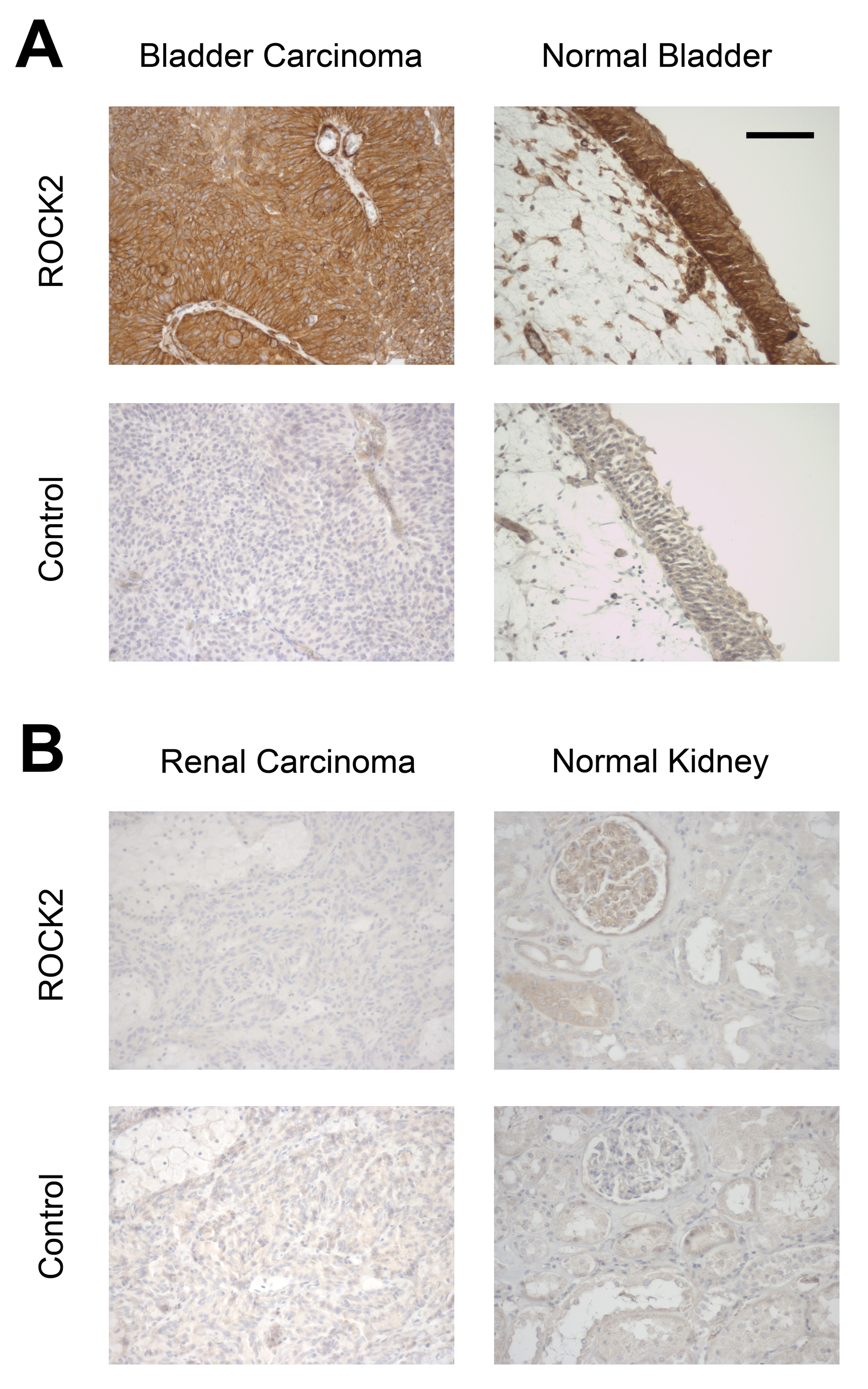

Supplement: Supplementary file 1 — Immunohistochemical staining of the patient’s tumor tissue. Immunohistochemical staining with polyclonal rabbit anti-ROCK2 antibody (1:250) reveals strong reactivity in the patient’s invasive bladder carcinoma as well as in normal tumor free urothelium (A). However, ROCK2 shows an only weak staining in normal glomerular Bowman’s epithelium and in proximal tubule epithelium whereas expression is absent from patient’s papillary renal cell carcinoma (B). Rabbit immunoglobulin control stainings are negative, as expected. (scale bar = 100 μm; all figures same magnification). (TIF 13993 kb) [file 40478_2017_447_MOESM1_ESM.tif]

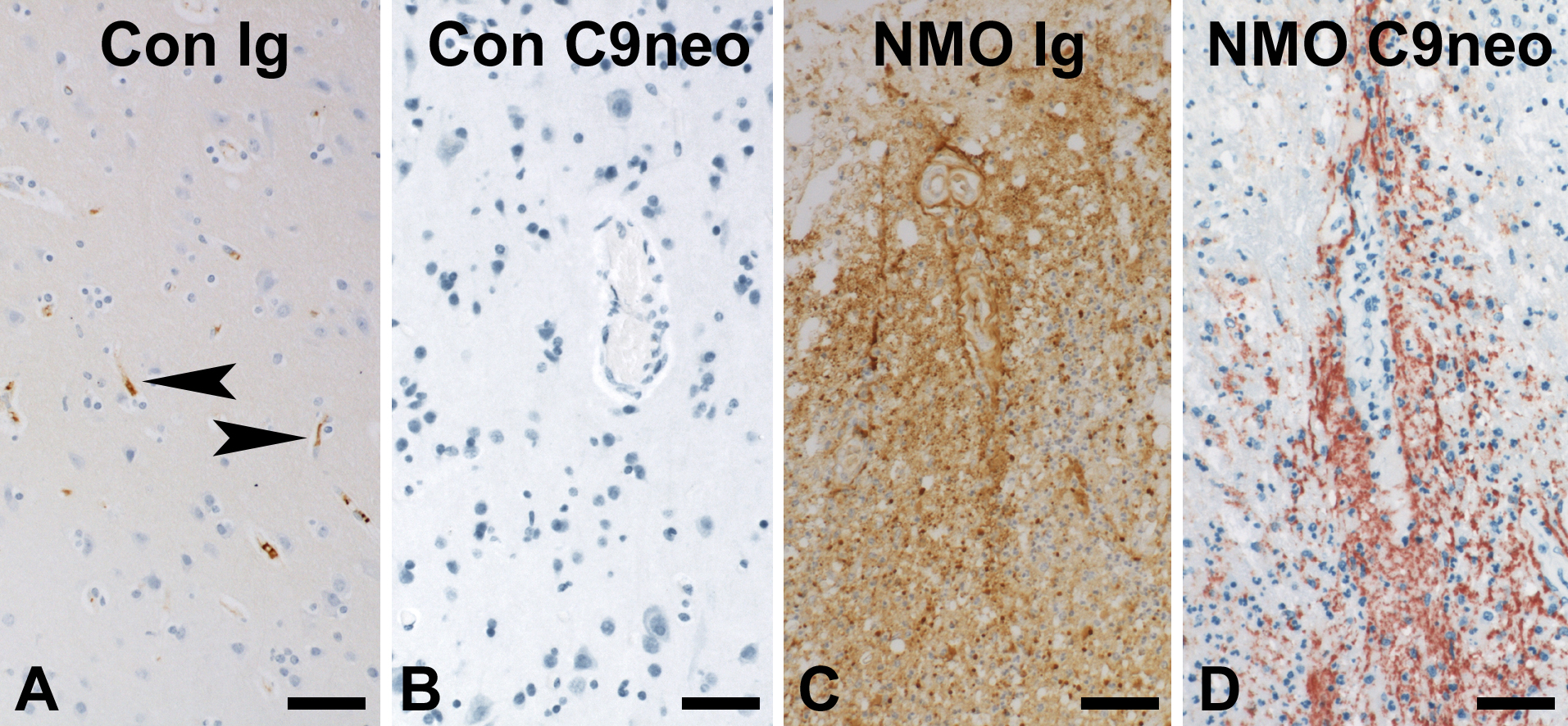

Supplement: Supplementary file 4 — Control stainings for Ig and C9neo. (A) Staining for Ig in control brain shows some reactivity in bloodvessels (arrowheads). Bar: 50 μm. (B) Staining for C9neo shows no reactiity in the brain. Bar: 50 μm. (C) Staining for Ig in the brain of an NMO patient shows strong deposition of Ig around a bloodvessel. Bar: 50 μm (D) Staining for C9neo, in this case shows deposition around a bloodvessel. (TIF 4965 kb) [file 40478_2017_447_MOESM4_ESM.tif]
