# Supplementary material for: Rho-associated protein kinase 2 (ROCK2): a new target of autoimmunity in paraneoplastic encephalitis
Source: Acta Neuropathol Commun. 2017 May 29;5:40. doi: 10.1186/s40478-017-0447-3 (PMC5448146; doi:10.1186/s40478-017-0447-3)
Supplement: Supplementary file 2 — Patients with urological cancers whose sera were screened for anti-ROCK2 antibodies. (DOCX 14 kb) [file 40478_2017_447_MOESM2_ESM.docx]

**Additional file 2: Table S1.** Patients with urological cancers whose sera were screened for anti-ROCK2 antibodies

| **Patient gender & age** | **Tumor** | **UICC TNM Staging** |
| --- | --- | --- |
| F 39 | Urothelial carcinoma of the bladder | T3b N0 M0 L1 V1 Pn0, Stage III, R0 |
| M 56 | Urothelial carcinoma of the bladder | pT4a pN1 M0 L1 V1 Pn0, Stage IV, R1 |
| M 86 | Urothelial carcinoma of the ureter | pT1 pNX M0 L0 V0 Pn0, Stage I, R0 |
| M 63 | Urothelial carcinoma of the bladder | pT4b pN2 M0 L1 V1 Pn1, Stage IV, R1 |
| M 59 | Urothelial carcinoma of the bladder | ypT1 pN0 M0 L0 V0 Pn0, Stage I, R0 |
| F 63 | Urothelial carcinoma of the bladder | ypT0 pN0 M0 L0 V0 Pn0, Stage 0, R0 |
| M 72 | Urothelial carcinoma of the bladder | rpT2b pN0 M0 L1 V0 Pn0, Stage II, R0 |
| M 80 | Urothelial carcinoma of the bladder | pT4 pN0 M0 L1 V1 Pn1 R2 G3 |
| F 74 | Urothelial carcinoma of the bladder and the ureter | pT3a pNX pM1 L1 V1 Pn1, Stage IV, R0 |
| M 71 | Urothelial carcinoma of the bladder and the urethra | Bladder: pT3a pNX pM1 L1 V1 Pn1, Stage IV, R0  Urethra: pT3a pNX pM1 L1 V1 Pn1, Stage IV |
| M 78 | Urothelial carcinoma of the bladder | ypT0 pN0 M0 L0 V0 Pn0, Stage 0, R0 |
| F 53 | Urothelial carcinoma of the bladder | pTis, Stage 0is, R0 |
| M 75 | Urothelial carcinoma of the bladder | rpT2b pN0 M0 L1 V1 Pn0, Stage II, R0 |
| M 83 | Urothelial carcinoma of the bladder | rT1 pN0 M0 L0 V0 Pn0, Stage I, R0 |
| M 65 | Urothelial carcinoma of the bladder and the urethra | Bladder: pT1 pN0 M0 L1 V0 Pn0, Stage I, R0. Urethra:pT2 pN0 M0 L0 V0 Pn0, Stage II, R0 |
| M 47 | Urothelial carcinoma of the bladder | ypT0 pN0 M0 L0 V0 Pn0, Stage 0 |
| M 66 | Urothelial carcinoma of the bladder | pT4a pN1 M0 L0 V0 Pn1, Stage IV |
| M 51 | Urothelial carcinoma of the bladder | pT2b pN0 M0 L1 V1 Pn0, Stage II, R0 |
| M 76 | Urothelial carcinoma of the bladder | pT1b pN0 M0 LX VX PnX, Stage I, R0 |
| M 65 | Urothelial carcinoma of the bladder | pT2a pN0 M0 L0 V0 Pn0, Stage II, R0 |
| F 54 | Renal cell carcinoma | pT1a pNX M0 L0 V0 Pn0, Stage I, R0 |
| F 77 | Renal cell carcinoma | pT3a pNX M0 L0 V0 Pn0, Stage III, R0 |
| F 84 | Renal cell carcinoma | pT3a pNX M0 L1 V2 Pn0, Stage III, R0 |
| M 62 | Renal cell carcinoma | pT2a pN0 M0 L1 V1 Pn1, Stage II, R0 |
| M 75 | Renal cell carcinoma | pT3a pN0 M0 L1 V2 Pn0, Stage III, R0 |
| M 72 | Renal cell carcinoma | rpT3a pNX M0 L1 V1 Pn0, Stage III, R0 |
| M 76 | Renal cell carcinoma | pT1b pNX M0 L0 V0 Pn0, Stage I, R0 |
| M 56 | Renal cell carcinoma | pT1b pNX M0 L0 V0 Pn0, Stage I, R0 |
| M 69 | Renal cell carcinoma | pT3a pNX M0 L1 V2 Pn0, Stage III, R0 |
| M 63 | Renal cell carcinoma | pT1b pNX M0 L0 V0 Pn0, Stage I, R0 |
| M 58 | Renal cell carcinoma | pT1b pNX M0 L0 V0 Pn0, Stage I, R0 |
| F 76 | Renal cell carcinoma | pT1b pNX M0 L1 V1 Pn0, Stage I, R0 |
| F 74 | Renal cell carcinoma | pT1b pN0 M0 L0 V0 Pn0, Stage I |
| M 65 | Renal cell carcinoma | pT1b pN0 M0 L1 V1 Pn0, Stage I, R0 |
| M 79 | Renal cell carcinoma | pT3a pN1 M0 L1 V0 Pn0 Stage III, R0 |
| M 57 | Renal cell carcinoma | pT1a pNX M0 L0 V0 Pn0, Stage I, R0 |
| M 58 | Renal cell carcinoma | pT1a pNX M0 L0 V0 Pn0, Stage I, R0 |

Abbreviations: F, female; M, male.
