# Supplementary material for: Rho-associated protein kinase 2 (ROCK2): a new target of autoimmunity in paraneoplastic encephalitis
Source: Acta Neuropathol Commun. 2017 May 29;5:40. doi: 10.1186/s40478-017-0447-3 (PMC5448146; doi:10.1186/s40478-017-0447-3)
Supplement: Supplementary file 3 — Supplementary Methods. Immunohistochemistry of brain biopsy; Mass spectrometry. Supplementary Results: Routine neural antibody testing. (DOCX 19 kb) [file 40478_2017_447_MOESM3_ESM.docx]

**Supplemental Data**

**METHODS**

**Immunohistochemistry of brain biopsy**

Paraffin sections containing formalin fixed brain biopsy material were stained for lymphocyte markers CD3 (Dako Glostrup, Denmark, A0452, 1:2000), CD8 (Labvision, Fremont, CA, MS-457, 1:2000) and granzyme B (GrB, Labvision, Fremont, CA, RB-9015, 1:1000), for CD68 (Dako Glostrup, Denmark, M0814, 1:100) recognizing macrophages and microglia as well as for immunoglobulin (biotinylated donkey anti-human, Jackson ImmunoResearch, West Grove, PA, 709-005-149, 1:200) deposits and C9neo (Gift from S. Piddlesden, Univ. of Cardiff, UK, 1:20), the terminal complex of the complement cascade. As negative control for Ig or complement deposition we used a control without any neurological disease. As positive control we used brain tissue from a neuromyelitis optica patient (see suppl. figure 2). Neurons were detected by NeuN (Millipore, Billerica; MA, USA, MAB377, 1:250). Stainings for CD3, CD8, GrB and ROCK2 were enhanced by using catalyzed system amplification with biotinylated-tyramide as described previously.^1^ Quantification of CD3^+^, CD8^+^, GrB^+^, and CD68+ cells was performed in 4 mm^2^ of tissue by using a morphometric grid. Terminal deoxynucleotidyl transferase-mediated dUTP nick-end labeling (TUNEL) staining was performed with the In Situ Cell Death Detection Kit (Roche, Basel, Switzerland) following the suppliers protocol. After autoimmunity against Rho-associated protein kinase 2 (ROCK2) was identified, we also stained for ROCK2 (HPA007459, Sigma-Aldrich) at a concentration of 1:250 as primary antibody. Confocal fluorescent triple immunostaining (NeuN/CD8/GrB or ROCK2/CD3/GrB) was done after sections were washed in 0.05 M PBS prior to heat-induced epitope retrieval (HIER) by heating in household food steamer device (MultiGourmet FS 20, Braun, Kronberg/Taunus, Germany) for 60 minutes at 100°C in citrate buffer (pH = 6.0). This was followed by sequential incubations with the primary antibodies. First, sections were incubated with anti-ROCK2 (1:5000) or NeuN (Millipore, Billerica, MA, USA, 1:1000). This was followed by incubation with biotinylated sheep anti-mouse (Jackson), followed by incubation with avidin-peroxidase (Sigma, Germany) and then again followed by catalyzed system amplification with biotinylated-tyramide. In order to inactivate the binding properties of the first round of antibodies and to retrieve additional CD8 and Granzyme-B epitopes, sections were then treated with EDTA (10 mM, pH = 9.0) in TRIS buffer for 45 minutes followed by washing and final incubation with avidin-Cy5 (Jackson Immunoresearch, West Grove, PA) for 1h. After this, sections are incubated with rabbit-anti-CD8 (Dako Glostrup, Denmark, 1:50) and mouse-anti-Granzyme-B (Labvision, Fremont, CA, 1:50) overnight. As secondary antibodies we used Cy2-conjugated donkey-anti-mouse (1:200) and Cy3-conjugated donkey-anti-rabbit (1:200) both from Jackson Immunoresearch.

**Mass spectrometry**

1. Mass spectrometry sample preparation was performed as reported by Koy et al.^2^ Unless otherwise indicated, hardware, software, MALDI targets, peptide standards and matrix reagents were obtained from Bruker Daltonics, Bremen, Germany. Briefly, samples were reduced with dithiothreitol and carbamidomethylated with iodoacetamide prior to SDS-PAGE. Proteins were visualized with Coomassie Brilliant Blue G-250 and visible protein bands were excised and destained. After tryptic digest peptides were extracted and spotted with α-cyano-4-hydroxycinnamic acid onto a MTP AnchorChip™ 384 TF target. MALDI-TOF/TOF measurements were performed with an Autoflex III smartbeam TOF/TOF200 System using flexControl 3.3 software. MS spectra for peptide mass fingerprinting (PMF) were recorded in positive ion reflector mode with 6000 shots and in a mass range from 700 Da to 4000 Da. Spectra were calibrated externally with the commercially available Peptide Calibration Standard II, processed with flexAnalysis 3.3 and peak lists were analyzed with BioTools 3.2. The Mascot search engine Mascot Server 2.3 (Matrix Science, London, UK) was used for protein identification by searching against the NCBI database limited to Mammalia. Search parameters were as follows: Mass tolerance was set to 80 ppm, one missed cleavage site was accepted, and carbamidomethylation of cysteine residues as well as oxidation of methionine residues were set as fixed and variable modifications, respectively. To evaluate the protein hits, a significance threshold of p<0.05 was chosen. For further confirmation of the PMF hits two to five peptides of each identified protein were selected for MS/MS measurements using the WARP feedback mechanism of BioTools. Parent and fragment masses were recorded with 400 and 1000 shots, respectively. Spectra were processed and analyzed as described above with a fragment mass tolerance of 0.7 Da.

1. Bauer J, Lassmann H. Neuropathological techniques to investigate central nervous system sections in multiple sclerosis. Methods Mol Biol 2016;1304:211-229.

2. Koy C, Mikkat S, Raptakis E, et al. Matrix-assisted laser desorption/ionization-quadrupole ion trap-time of flight mass spectrometry sequencing resolves structures of unidentified peptides obtained by in-gel tryptic digestion of haptoglobin derivatives from human plasma proteomes. Proteomics 2003;3:851–858.

**RESULTS**

**Routine neural antibody testing**

Serum and CSF were negative for antibodies against the following antigens (protocols reported elsewhere^1^): Hu, Ri, Yo, CV2, amphiphysin, Ma2, glutamic acid decarboxylase 65 kD isoform (GAD65), Sox1, Delta/Notch-like EGF-related receptor (DNER) Zic4, (immunoblots, Ravo, Freiburg/Germany), N-methyl-D-aspartate (NMDA) receptor, leucine-rich glioma inactivated protein 1 (LGI1), contactin-associated protein-2 (CASPR2), Glycine receptor, α-amino-3-hydroxy-5-methyl-4-isoxazolepropionic acid (AMPA) receptor, γ-aminobutyric acid (GABA) B receptor, metabotropic glutamate receptor (mGluR) 5, dipeptidyl-peptidase-like protein-6 (DPPX), GAD65 (fixed cell-based assays, Euroimmun, Lübeck/Germany).

1. Dogan Onugoren M, Deuretzbacher D, Haensch CA, et al. Limbic encephalitis due to GABAB and AMPA receptor antibodies: a case series. J Neurol Neurosurg Psychiatry 2015;86:965-972.
